# Application of Smart Watch-Based Functional Evaluation for Upper Extremity Impairment: A Preliminary Study on Older Emirati Stroke Population

**DOI:** 10.3390/s25051554

**Published:** 2025-03-03

**Authors:** Yeo Hyung Kim, Sarah Kim, Hyung Seok Nam

**Affiliations:** 1 Department of Rehabilitation Medicine, College of Medicine, The Catholic University of Korea, Seoul 06591, Republic of Korea; drkyh@catholic.ac.kr; 2 Department of Rehabilitation Medicine, Sheikh Khalifa Specialty Hospital, Ras al Khaimah 6365, United Arab Emirates; 3 Department of Rehabilitation Medicine, Seoul National University Hospital, Seoul 03080, Republic of Korea

**Keywords:** smartwatch, wearable sensor, rehabilitation, stroke, functional evaluation, motion segment size

## Abstract

Smartwatch-based functional assessments for upper extremity movement are a promising tool for a detailed and serial assessment during stroke rehabilitation, but their clinical application remains challenging. In this study, nine patients with hemiparesis due to a stroke participated in occupational therapy sessions using virtual reality-based rehabilitation devices. An Action Research Arm Test (ARAT) was performed at baseline and after intervention, with wrist smartwatch sensors recording motion data. We extracted acceleration and gyro sensor data from smartwatches and calculated the average motion segment size (MSS) as a measure of motion smoothness. Among the included patients, four participants completed all 10 therapy sessions and the follow-up evaluation. The average MSSs of acceleration for all *x*, *y*, and *z* directions were significantly correlated with the ARAT scores across all task domains. For angular motion, the average MSS in the gross movement task (domain 4) showed strong correlations with the ARAT scores: roll (r_s_ = 0.735, *p* = 0.004), pitch (r_s_ = 0.715, *p* = 0.009), and yaw (r_s_ = 0.704, *p* = 0.007). At the serial follow-ups, most participants showed a considerable increase in the average MSSs of the roll, pitch, and yaw angles measured during domain 4, alongside improvements in their clinical ARAT scores. Our findings support the feasibility of using commercial smartwatch-based parameters for upper extremity functional evaluations during stroke rehabilitation and highlight their potential for serial follow-up assessments.

## 1. Introduction

The application of wearable sensors has become increasingly widespread in healthcare, particularly in assessing physical activities in the field of rehabilitation [1]. Over the past decade, extensive research has been conducted utilizing inertial measurement unit (IMU) sensors in both laboratory and clinical settings [2]. Initially, lab-designed sensor acquisition systems were predominantly used; however, commercial sensor systems for human motion analyses have gained prominence in recent years [3]. Additionally, sensors with long-term battery capabilities such as ActiGraph are now employed to monitor physical activities in daily life [4]. Also, in the context of long-term data acquisition, utilizing energy harvester devices is also being investigated [5]. While some research outcomes from these sensors have been significant and relevant to healthcare, universally accepted and widely usable sensor systems, along with standardized parameters, remain scarce. This is primarily due to the variability in the sensor systems used by researchers and practical challenges such as wearability and setup difficulties.

Recent advancements in smartphone and smartwatch technology have further expanded the scope of wearable sensor applications, enabling the real-time monitoring of various physiological and biomechanical parameters, including step count, heart rate, electrocardiograms, and respiratory metrics—for healthcare monitoring and assessment [6]. In particular, smartwatches have emerged as an accessible and cost-effective alternative for continuous health monitoring, with embedded accelerometers and gyroscopes for motion tracking. However, despite their widespread availability, leveraging smartwatch data for clinical assessments remains a challenge. Raw sensor data do not directly reflect clinical function, requiring data processing and the identification of relevant clinical outcome measures [7].

Motion detection, which often relies on machine learning algorithms, has emerged as a widely utilized approach; however, real-time wrist motion tracking remains an active area of research [8,9,10]. Motion characteristics are also significant features of upper extremity movements in patients with functional impairments during activities of daily living (ADL) [11]. Although metrics such as jerk and the spectral arc of sensor data have been used to evaluate motion smoothness [12], they are insufficient to fully represent clinical conditions. A recent study proposed kurtosis as an outcome measure to assess upper extremity movement diversity [13]. Despite these advancements, the application of smartwatches in rehabilitation settings remains limited due to the lack of standardized assessment protocols and clinically validated outcome measures.

Several studies have highlighted the application of smartwatches in rehabilitation assessments and programs. A study performed on Parkinson’s disease investigated the use of a smartwatch to detect the activities of daily living, demonstrating their potential for long-term home-based disease-specific symptom tracking [14]. Similarly, research has shown the effectiveness of self-directed rehabilitation paired with a smartwatch in a few neurological and musculoskeletal disorders [15,16]. Efforts to evaluate and monitor upper limb function in stroke patients using smartwatch-based accelerometry data have been attempted in a limited number of pilot studies [17,18,19]; however, objective and notable results have not been achieved yet. Moreover, variations in smartwatch models, sensor specifications, and data processing algorithms may result in inconsistencies in the outcomes. Addressing these challenges requires standardized protocols for data collection and analysis, as well as extensive validation studies across various etiologies and populations.

Clinical functional evaluation plays a critical role in the rehabilitation of patients with neurological disorders, such as a stroke or spinal cord injury, which impair the movements and activities of daily living (ADL) [20]. Comprehensive functional evaluations are essential for effective rehabilitation planning and monitoring progress. Traditionally, tools such as the Action Research Arm Test (ARAT), Wolf Motor Function Test (WMFT), and Modified Barthel Index (MBI) have been widely used [21,22]. However, these measurement tools are labor-intensive, time-consuming, and often fail to capture the critical aspects of impaired movements. Wrist-worn smartwatches offer promising potential for upper extremity functional evaluations during rehabilitation by providing sensor-based data; yet, their application remains limited to basic metrics. To address this gap, we introduced a novel outcome measure, motion segment size (MSS), which quantifies movement smoothness and magnitude along anatomical axes using smartwatch sensors [23]. In a pilot study involving Korean patients with musculoskeletal injuries of the upper extremities, MSS demonstrated consistent results with clinical function despite a small sample size [24].

In this study, we aimed to evaluate the feasibility of the previously developed sensor-based parameters based on a single wrist smartwatch, MSS, for assessing upper extremity function in stroke patients with hemiparesis. To explore its potential applicability across diverse populations, this study focused on a different ethnic group, specifically the Emirati population, compared to the prior research. Additionally, this study sought to investigate the suitability of MSS for serial follow-up evaluations.

## 2. Materials and Methods

### 2.1. Study Protocol and Participants

This study evaluated upper extremity functions by both clinical functional evaluation and a smartwatch measurement before and after 10 occupational therapy sessions over a 2–3 week period. The occupational therapy used virtual reality-based rehabilitation devices (Rapael Smart Board^TM^ and Smart Glove^TM^, Neofect Inc., Yong-in, Republic of Korea) to facilitate functional improvement as much as possible [25,26]. This device is a task-specific, interactive, and game-based virtual reality rehabilitation system that uses a soft glove or an end-effector type moving board which provides various ADL tasks, facilitating neuroplasticity, which has led to greater motor recovery and higher motivation levels in several studies [27].

**Figure 1:**
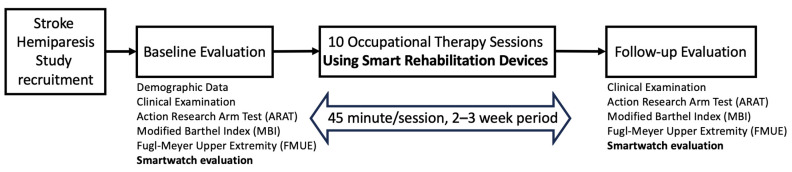
Flow diagram of the clinical study protocol is shown.

Nine patients with hemiparesis due to a stroke that resulted in the functional impairment of a unilateral upper extremity were enrolled in this study between March 2022 and October 2023 in a tertiary hospital specializing in neuroscience. The inclusion criteria were a diagnosis of a stroke that impaired unilateral upper extremity function, and the ability to follow the therapist’s instructions. The exclusion criteria were having moderate to severe cognitive impairments (Mini-mental Status Exam (MMSE) score 20 or less) [28], no voluntary movements of the upper extremities, and severe spasticity (modified Ashworth Scale score of 3 or higher). The patients were screened by a rehabilitation consultant physician and enrolled based on the inclusion and exclusion criteria. Among the included patients who performed the baseline evaluation, four patients completed the 10 therapy sessions and follow-up evaluations. A flow diagram of this study is shown in Figure 1. All participants provided written informed consent. The study protocol was approved by the Institutional Review Board (MOHAP/DXB-REC/O.JF/No.111/2021) of a tertiary hospital.

### 2.2. Clinical Functional Evaluation

For each evaluation session separately performed before and after the 10 therapy sessions, demographic data including age, sex, diagnosis and onset date of the stroke, hemiparesis laterality, and the evaluation date were recorded. For the clinical functional assessment, the Action Research Arm Test (ARAT) [29], modified Barthel Index (MBI) [22], and Fugl-Meyer Assessment Score Upper Extremity (FMUE) [29] were evaluated.

### 2.3. Smart Watch-Based Data Acquisition and Processing

After the clinical functional evaluations, the participants wore two Galaxy Watch^®^ devices (Samsung Electronics Co., Ltd., Suwon, Republic of Korea) on each wrist. The setting and data acquisition methods were consistent with those used in our previous research, and detailed information was provided in earlier publications [23,24]. In brief, the participants were asked to perform each task set separately after calibration (by pressing the button on the data logger application) each time to minimize the signal draft, while in each task set, they were told to perform the tasks in order, continuously, following the therapist’s guidance. Most of the participants were familiar with the task sets because a clinical evaluation of the ARAT was performed just before sensor data recording. Sensor data recordings were separately performed for the 4 domains of the ARAT: (1) grasping wooden blocks and reaching, (2) moving items on a table, (3) pinching marbles and reaching, and (4) gross movements requiring the lifting of an arm [23]. The smart watch IMU sensor data were acquired at an average sampling rate of 25Hz and transferred through the Bluetooth^®^-linked data logger application. The sampling rate may be lower than usual for a motion analysis; however, it was shown in the literature that 25 Hz and 100 Hz accelerometer data in a physical activity analysis had a significant correlation [30].

### 2.4. Sensor-Based Parameters

**Figure 2:**
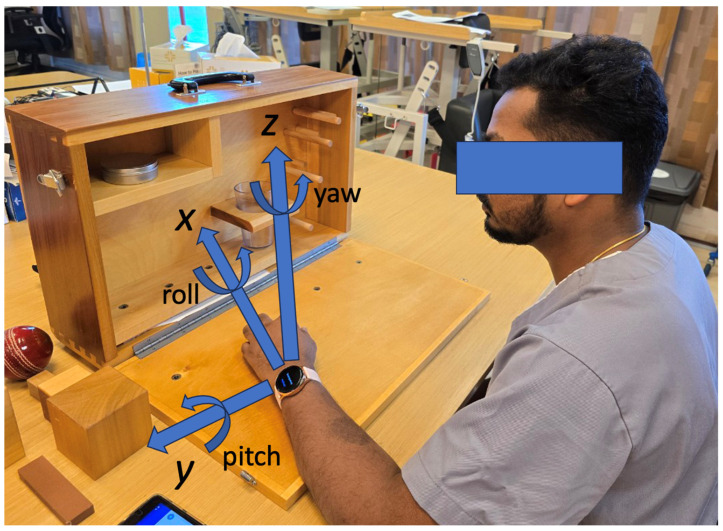
A person is wearing a smartwatch in the calibrating posture determined to be sitting on a chair with an arm put naturally on the desk in an elbow-flexed and forearm-pronated posture position. The orthogonal coordination and the gyro sensor axes are shown, where the roll, pitch, and yaw angles are assumed to represent the anatomical movements of forearm pronation/supination, elbow flexion/extension, and shoulder internal/external rotation, respectively.

The orthogonal coordinate system used in our previous study was applied to the current study’s data acquisition [24], with the calibrating posture set as sitting on a chair with an arm put on the desk in an elbow-flexed and forearm-pronated posture position (Figure 2). The *x*- and *y*-axes represented the planar direction, and the *z*-axis represented the vertical direction. The angular data for roll, pitch, and yaw were also collected by applying the integration matrix to the angular velocity followed by a rotation matrix, in respect to the calibration position and direction. As in our previous study [24], we assumed that the roll, pitch, and yaw angles of the wrist-worn smartwatch data represented the anatomical movement of forearm pronation/supination, elbow flexion/extension, and shoulder internal/external rotation, respectively, although in reality all joint movements contribute simultaneously. The MSSs for all accelerations and angles were calculated in each task domain as well as for the performance time (in seconds) [23]. MSS is defined as the size of a positional or angular movement in the same direction before changing the direction, calculated as Equation (1) [23]:

(1) $$\mathrm{MSS}=\sum_{t=i}^{i+1}\left|data\left(t\right)-data\left(t-1\right)\right|$$

where *i* is the dataset of timepoints where data′ (*i*) = 0. The average MSS was calculated for all motion segment values exceeding 10° for angles and 0.05 m/s^2^ for acceleration [23]. All data processing was performed using the analysis program built on MATLAB R2024b software (The Mathworks, Inc., Natick, MA, USA).

### 2.5. Serial Follow-Up of Sensor-Based Parameters

For a novel parameter to be practically used in clinics, it must demonstrate significant changes corresponding to alterations in the patient’s functional status, with values distributed within an appropriate range [23,24,31,32]. To assess the clinical applicability of our sensor-based parameters, we present the individual data as a case series for the participants who completed the study protocol, including the follow-up assessments. We chose the case series approach to experimentally explore whether changes in MSS occur during a 2–3 week rehabilitation period in stroke patients. Although the small number of participants and the absence of a control group limit the feasibility of a statistical analysis, this approach would provide valuable longitudinal insights that will serve as a foundation for future large-scale studies. If serial changes in MSS can be reliably detected, they may provide a more objective and personalized assessment of rehabilitation progress.

### 2.6. Statistical Analysis

For the correlation analysis between the sensor-derived parameters and clinical functional evaluation scores, Spearman’s correlation analyses were performed using SPSS 28.0 (IBM, Armonk, NY, USA). The *p* values less than 0.05 were considered to be statistically significant.

## 3. Results

### 3.1. Demographic Data

Nine participants (five men and four women) with unilateral hemiplegia due to a stroke were enrolled in this study. The mean age was 61.3 ± 14.5 years old. The median duration since stroke onset was 28 days. Among the participants, four of them completed the study protocol and had a follow-up evaluation. Demographic data of the study participants are shown in Table 1.

### 3.2. Correlation Analysis Between Average MSS and ARAT Score

**Figure 3:**
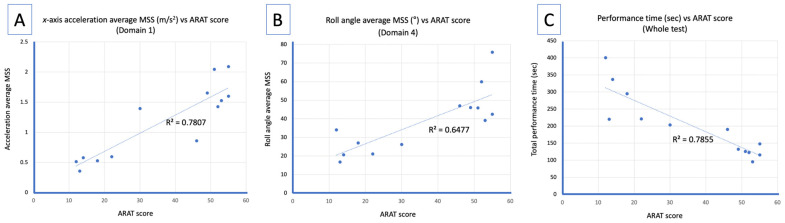
Representative sensor-based parameters demonstrating a strong correlation with Action Research Arm Test (ARAT) scores are shown for (**A**) *x*-axis acceleration average motion segment size (MSS) for domain 1, (**B**) roll angle average MSS for domain 4, and (**C**) total performance time of the whole test. The linear regression line and R^2^ values are shown for each parameters.

Table 2 shows the results of the correlation analysis between the smartwatch-based parameter (average MSS) and clinical functional scale score (ARAT) using the dataset acquired from this study (n = 13 including follow-up data collection). The ARAT score showed a significant correlation with the FMUE (r_s_ = 0.938, *p* < 0.001), MBI (r_s_ = 0.812, *p* < 0.001), and total performance time (r_s_ = −0.891, *p* < 0.001). For the sensor-based parameters, the average MSSs of acceleration for all x, y, and z directions were significantly correlated with the ARAT scores in all four domains and also the whole test. For the angular data, the average MSSs for all the parameters in domain 4 (r_s_ = 0.735, *p* = 0.004 for roll; r_s_ = 0.715, *p* = 0.009 for pitch; and r_s_ = 0.704, *p* = 0.007 for yaw) showed a significant correlation with the ARAT scores, whereas in the whole test dataset only the roll and pitch angles (r_s_ = 0.754, *p* = 0.003 for roll; r_s_ = 0.688, *p* = 0.009 for pitch; and r_s_ = 0.465, *p* = 0.109 for yaw) showed significance. In domain 3, the pitch angle showed a significant correlation with the ARAT score (r_s_ = 0.671, *p* = 0.012). Detailed data are shown in Table 2 and Figure 3.

### 3.3. Case-Series of Average MSS Before and After the Intervention

Table 3 presents detailed longitudinal data on the smartwatch-derived parameters (average MSS) and clinical functional scale scores (ARAT) for the four participants who completed the intervention through 10 sessions of occupational therapy. Although a statistical analysis is practically not applicable, all the participants showed improvement in their ARAT scores, especially subject 1 who experienced a stroke most recently, had moderate functional impairment, and showed the greatest improvement. All participants showed an approximately 10-point increase in their FMUE scores.

Regarding the sensor-derived parameters, three participants showed an increase in the average MSSs of the roll, pitch, and yaw angles measured during domain 4 of the ARAT task, and improvement in total performance time. Subject 5 showed a decrease in such parameters despite an improvement in their functional score. The mean changes in the average MSSs were 11.5 ± 14.2, 9.1 ± 1.5, and 24.1 ± 27.9 degrees for the roll, pitch and yaw angles, respectively.

## 4. Discussion

This study aimed to assess the feasibility of the newly proposed sensor-based parameter, average MSS, as an indicator of clinical function in Emirati patients with a history of a stroke [24]. In contrast to our previous study, the participants in this study were hemiparetic patients with stroke histories, and the participants were from a completely different ethnic background. The average MSS for the acceleration and angular data obtained from the commercial smartwatches demonstrated significant correlations with the clinical functional evaluation data in the present study. Gross movements involving overhead activities showed a better correlation in the angular data. Therefore, our research, as a pilot study, suggests that average MSS could be applicable to neurologically impaired patients, including those with a history of a stroke, and across various populations. Furthermore, the meaningful changes observed in the average MSS during the serial follow-up evaluations before and after the intensive rehabilitation sessions suggest that the motion data obtained from the smartwatches have the potential to be applied as an efficient method for monitoring clinical changes during rehabilitation [33].

From the correlation analysis between the average MSS data and the ARAT scores, the acceleration average MSS in all orthogonal directions in each domain and the whole test showed a correlation of considerably high significance. This result is generally in line with the findings of the previous study performed with an IMU sensor-based motion analysis system [23], where the average MSS of acceleration in all x, y, and z directions for hand sensors during the full ARAT test showed a significant correlation with the ARAT score. Also, when compared to the results of the previous study performed with a single smartwatch, it is consistent in that the average MSS for the z-direction in gross ADL tasks and the average MSS for the x- and y-directions during the planar tasks showed significant correlations with the ARAT scores [24]. Saes et al. [34] used the spectral arc length (SPARC) method to assess planar movement velocity during a reach-to-grasp task in stroke patients and demonstrated that SPARC is highly correlated with the FMUE score and also potentially applicable in serial measurements. It shows similar aspects of the upper extremity movement; however, it is a dimensionless, calculated parameter where intuitive clinical meaning is limited. There are many other methods such as jerk and the number of peaks that assess motion smoothness [35], but up to now velocity-related parameters seem to be the most widely investigated methods for post-stroke functional upper extremity assessments [36].

The novel feature of the current study is that we utilized a commercial smartwatch on a wrist to evaluate movement quality and smoothness, in the relation to anatomical joint movement. To our knowledge, this is the first attempt to perform such an evaluation on post-stroke patients. Using a single sensor means that we are not aiming to achieve accuracy in the motion analysis, but to find a simple and reliable parameter that correlates with functional status [37,38]. When using a multi-sensor system, the proximal sensor acts as a reference sensor to determine the motion of the distal sensor in each segment, resembling a human joint system (i.e., body trunk–shoulder–elbow–wrist) with accurate positions [39]. A single-sensor system lacks a reference sensor, so motion reconstruction would result in non-unique solutions. However, the human joint system is also complex with a significantly high degree of freedom [40]. In this context, it can be assumed that the motion characteristics of the end-organ (hand) are more relevant to clinical functional status. To meet this requirement, the movements that are challenging but improvable in stroke patients should be selected for data acquisition, which makes clear a signal change in the sensors when the subject performs them depending on the level of impairment [41]. In this study, our methodology focused on extracting angular data in three axes, roll, pitch and yaw, by assuming that these movements reflect anatomical joint axes such as forearm supination/pronation, elbow flexion/extension, and shoulder internal/external rotation. The results demonstrated a significant correlation between the ARAT score and the average MSS of all roll, pitch, and yaw angles in domain 4 tasks and roll/pitch angles in the whole test. This is generally consistent with our previous studies that showed the roll and pitch angles average MSS during the ARAT domain 4 tasks and also during the whole test had a significant correlation with the ARAT scores with appropriate variation across disease severity [23,24]. In the current study, the yaw angle also showed good correlation in domain 4. Also, the pitch angle in domain 3 showed significant correlation with the clinical measure. This is also supported by our previous result that showed the data collected during the domain 3 ARAT specifically presented a high correlation with the pitch angle [23]. In addition, performance time consistently showed a significant correlation with clinical functional status across all studies performed until now [23,24].

One of the most important features of a newly suggested parameter in order for it to be widely accepted is that it needs to present a consistent value range in different location or population settings. In our initial study, the roll (forearm supination/pronation axis) angle average MSS during ARAT domain 4 tasks ranged from approximately 11 to 40 degrees depending on the impairment, from severe to nearly normal [23], whereas in the next study it ranged from approximately 25 to 45 degrees [24]. In the current study, it ranged from 17 to 60 degrees, while in one case in the post-intervention assessment it increased to 76 degrees. The results show that the average MSS values show a similar range in different experimental settings and across different ethnicities, with increasing values accompanying a better functional status. While the number of subjects in this study is not sufficient to determine the thresholds between the mild, moderate, and severe levels of impairment, considering the average MSS of the roll angle during the domain 4 ARAT, it appears that an average MSS of 30 or lower represents moderate to severe functional impairment whereas an average MSS of 40 or higher reflects mild functional impairment [42]. Further study with a larger sample size is necessary to present a more precise range.

In the presented case series (refer to Table 3), the intervention using the smart rehabilitation devices was intended to maximize neuroplasticity by providing repetitive task-oriented exercises [43]. Subject 1 was in the subacute phase with moderate functional impairment, and showed the most improvement in both the clinical and sensor-based assessments. This finding for subject 1 is consistent with previous studies indicating that early intervention yields more substantial benefits compared to individuals with chronic stroke. Patients in the early post-stroke phase may exhibit enhanced neuroplasticity and a greater responsiveness to interventions due to heightened neurobiological processes, such as synaptic remodeling and spontaneous recovery mechanisms [44,45]. Subject 2 was in the late subacute stage with severe impairment; however, their clinical score slightly improved (+4 points) whereas the average MSS for all angles also showed a considerable increase, along with a decreased performance time. Subject 5 was in the chronic stage, almost 1 year after the stroke onset, and demonstrated different results. Although the ARAT score increased by 6 points after the intervention, the average MSS values did not increase much and even decreased in some axes. This may be affected by other factors such as spasticity, and also compensatory movement habits that the patient had already developed [46]. However, it showed that intensive training using a smart rehabilitation device can improve function even in the chronic stage. Subject 6 had mild impairment in the acute stage, so after the treatment their movements seemed to have recovered to near normal motion considering the large increase in the average MSS value. These case series results imply that the average MSS values from the smartwatches may be used as an outcome measure for serial follow-ups for acute to subacute stroke rehabilitation.

The correlation coefficient values for the significant parameters shown in Table 2 range from approximately 0.63 to 0.89, with parameters such as acceleration average MSS in the x and z directions and total performance time showing 0.86, 0.84, and -0.89, respectively. Considering the small sample size, this still indicates strong agreement with the ARAT scores [47]. At this correlation level, we may assume that the average MSS derived from smartwatch data can be reliably used as an index for upper extremity function with at least more than 85% confidence. More samples are necessary to estimate an accurate level of confidence.

Considering the purpose of this study is to develop a commercial smartwatch-based functional evaluation method for practical clinical use, the most important advantage of the suggested method is its simplicity and the low-cost of the devices. Compared to multiple IMU sensor motion analysis systems, it significantly decreased the time for wearing and calibration as the patient just needs to wear the watch on their wrist and calibration is performed by pressing a button in a natural posture [23]. Moreover, due to its simplicity, serial or a daily evaluation is also possible by wearing it during the therapy sessions without spending extra time. Also, medical devices in the market are usually high-priced, while the suggested method is significantly cost-effective, requiring only a commercial smartwatch which costs approximately USD 200 and a smartphone or tablet application.

This study has several limitations. First, the number of participants is small, and it may be difficult to generalize the results to a different population. However, the results are supported by their consistency with the previous studies in similar settings, performed with different device sets (multi-sensor system vs. single smartwatch) or different disease entities (musculoskeletal disease vs. stroke). It is recommended that future studies expand the sample size to enhance the universality and reliability of the results. Second, the number of participants who completed the study protocol was also small. The reasons the participants did not complete the protocol were an unexpected early discharge from the hospital for inpatients, and poor compliance for outpatients. Initially, there was a control group, but the two participants allocated to the control group did not complete the protocol; therefore, only case series analyses for the intervention group using the smart devices could be performed to explore the potential utilization of a smartwatch for serial evaluations during stroke rehabilitation. A further study with a larger number of subjects should be conducted as a randomized controlled study. Fortunately, the completed cases varied in terms of the severity of the functional impairments and also the duration since stroke onset, so a meaningful interpretation could be performed. In addition, in a larger sample size study, patients with various etiologies for impaired upper arm function including other neurological or musculoskeletal conditions could be enrolled. In this regard, planar movement and overhead motion (similar to domain 4 tasks) may also show decreased smoothness due to pain and weak motor power in such patients [48]. This was partially demonstrated in the previous study [24], but a study with a larger number of subjects is necessary to generalize the findings. Lastly, the reliability of commercial smartwatches in the medical field is not fully established across various brands. While this issue needs to be addressed further in future studies, we assumed that the raw IMU data would be similar at a preliminary study level, as in the comparison study by Fuller et al. [49] showing that the step count data were similar across various commercial smartwatches.

## 5. Conclusions

A smartwatch-derived novel parameter, average MSS, showed significant correlations with clinical functional measures in Emirati hemiparetic patients with a history of a stroke. These study findings support the feasibility of using commercial smartwatch-based parameters as an index for upper extremity function in patients with hemiparesis due to a stroke. The results of this longitudinal case series highlight its potential for serial follow-up assessments during the course of intensive stroke rehabilitation.

## Figures and Tables

**Table 1:** Demographic data of the study participants (N = 9).

Subject No.	Age	Sex	Diagnosis	Days Since Onset	Hemiplegic Side	Initial Score			Follow-Up Score (After Intervention *)		
						ARAT	MBI	FMUE	ARAT	MBI	FMUE
1	42	M	Left pontine infarction	28	Right	30	77	44	53	93	55
2	63	F	Right basal ganglia, thalamic hemorrhage	148	Left	14	44	29	18	48	38
3	65	M	Left corona radiata, basal ganglia infarction	185	Right	46	80	56			
4	69	M	Left corona radiata, basal ganglia infarction	17	Right	12	17	16			
5	45	M	Left PCA infarction	344	Right	49	63	52	55	66	62
6	52	F	Right thalamic infarction	16	Left	51	83	53	55	92	62
7	56	F	Right MCA infarction	86	Left	52	86	56			
8	71	F	Left thalamic hemorrhage	25	Right	13	20	4			
9	89	M	Left middle cerebral artery territory infarction	26	Right	22	16	31			

PCA: posterior cerebral artery; MCA: middle cerebral artery; ARAT: Action Research Arm Test; MBI: modified Barthel Index; FMUE: Fugl-Meyer Upper Extremity. * For participants who completed the 10 treatment sessions with virtual reality-based rehabilitation devices.

**Table 2:** Correlation between smartwatch-based parameter average motion segment size (MSS) and Action Research Arm Test (ARAT) score.

ARAT Domain	*x*	*y*	*z*	Roll	Pitch	Yaw	Performance Time
1	0.894 ** (<0.001)	0.743 ** (0.004)	0.759 ** (0.003)	0.077 (0.802)	0.420 (0.175)	−0.357 (0.255)	−0.867 ** (<0.001)
2	0.814 ** (<0.001)	0.702 ** (0.008)	0.696 ** (0.008)	0.311 (0.301)	0.364 (0.245)	0.182 (0.572)	−0.757 ** (0.003)
3	0.682 * (0.010)	0.726 ** (0.005)	0.834 ** (<0.001)	0.393 (0.184)	0.671 * (0.012)	0.239 (0.431)	−0.520 (0.069)
4	0.635 * (0.020)	0.726 ** (0.005)	0.801 ** (0.001)	0.735 ** (0.004)	0.715 ** (0.009)	0.704 ** (0.007)	−0.713 ** (0.006)
Whole Test	0.856 ** (<0.001)	0.765 ** (0.002)	0.842 ** (<0.001)	0.754 ** (0.003)	0.688 ** (0.009)	0.465 (0.109)	−0.891 ** (<0.001)

Values are Spearman’s correlation coefficient (*p*-value). * Correlation is significant at the 0.05 level. ** Correlation is significant at the 0.01 level.

**Table 3:** Change of Action Research Arm Test (ARAT) score and Average Angular Motion Segment Size (MSS) values of four participants before and after treatment intervention.

Subject	ARAT Score	∆ARAT Score	∆FMUE Score	Domain 4 (Degrees) Average MSS			Total Time (s)
				Roll	Pitch	Yaw	
1	30→53	+23	+11	26.3→39.2	36.6→45.9	54.1→81.8	203→96.3
2	14→18	+4	+9	20.8→27.2	24.0→32.0	49.0→62.8	337→295
5	49→55	+6	+10	46.2→42.6	38.2→46.0	93.2→87.5	133→149
6	51→55	+4	+9	45.9→76.0	33.3→44.4	59.4→120	126→117

FMUE: Fugl-Meyer Upper Extremity.

## Data Availability

The dataset available on request from the authors.

## Abbreviations

The following abbreviations are used in this manuscript:

IMU	Inertial Measurement Unit
ADL	Activities of Daily Living
ARAT	Action Research Arm Test
MBI	Modified Barthel Index
MSS	Motion Segment Size
MMSE	Mini-mental Status Exam
FMUE	Fugl-Meyer Upper Extremity
